# Mutant KRAS Heterogeneity Shapes Nuclear Architecture During Pancreatic Cancer Initiation

**DOI:** 10.3390/epigenomes10010019

**Published:** 2026-03-10

**Authors:** Gareth Pollin, Angela J. Mathison, Elise N. Leverence, Thiago Milech De Assuncao, Juan Iovanna, Johnny C. Hong, Michael T. Zimmermann, Raul Urrutia, Gwen Lomberk

**Affiliations:** 1 Linda T. and John A. Mellowes Center for Genomic Sciences and Precision Medicine, Medical College of Wisconsin, Milwaukee, WI 53226, USA; g-pollin@outlook.com (G.P.); amathison@mcw.edu (A.J.M.); eleverence@mcw.edu (E.N.L.); tmilech@mcw.edu (T.M.D.A.); mtzimmermann@mcw.edu (M.T.Z.); 2 Division of Research, Department of Surgery, Medical College of Wisconsin, Milwaukee, WI 53226, USA; 3 Centre de Recherche en Cancérologie de Marseille (CRCM), Institut National de la Santé et de la Recherche Médicale (INSERM) U1068, CNRS UMR 7258, Aix-Marseille Université and Institut Paoli-Calmettes, Parc Scientifique et Technologique de Luminy, 13288 Marseille Cedex 09, France; juan.iovanna@inserm.fr; 4 Department of Solid Organ Transplantation and Hepatobiliary Surgery, Bon Secours Mercy Health St. Vincent Medical Center, Toledo, OH 43608, USA; johnnyhong5@gmail.com; 5 Department of Biochemistry, Medical College of Wisconsin, Milwaukee, WI 53226, USA; 6 Data Science Institute, Medical College of Wisconsin, Milwaukee, WI 53226, USA; 7 Department of Pharmacology and Toxicology, Medical College of Wisconsin, Milwaukee, WI 53226, USA; 8 Department of Molecular Pharmacology and Experimental Therapeutics, Mayo Clinic, Phoenix, AZ 85054, USA

**Keywords:** pancreatic ductal adenocarcinoma (PDAC), KRAS, variant heterogeneity, nuclear architecture, chromatin, phospho-proteomics, nucleolar organization, spliceosomal organization

## Abstract

Background/Objectives: Pancreatic ductal adenocarcinoma (PDAC) arises predominantly from activating KRAS mutations, yet individual genetic variants differ markedly in signaling output and clinical impact. G12D, the most prevalent variant, strongly drives oncogenic programs, whereas G12R signals less efficiently through the AKT and ERK pathways and is associated with longer patient survival than G12D-driven PDAC. Methods: To elucidate how these differences influence early cellular transformation, we expressed a panel of KRAS mutants in non-cancerous pancreatic ductal epithelial cells as a model of early PDAC initiation and profiled transcriptional and phospho-proteomic responses. We next examined whether epigenetic differences translate into mutation-specific changes in nuclear organization using quantitative imaging of G12D- and G12R-expressing nuclei at 24 and 48 h. Results: Each variant established a unique regulatory program enriched for chromatin remodelers, histone modifiers, and nuclear structural factors, indicating that variant-specific KRAS signaling rapidly develops divergent epigenetic states. Integrated transcriptomic and phospho-proteomic analyses identified G12D and G12R as the most divergent variants. G12D induced pronounced nuclear remodeling, including increased nuclear size, irregular morphology, and reorganization of the nucleolus and spliceosome, consistent with extensive chromatin and transcriptional reprogramming. In contrast, G12R elicited a weaker response, with minimal or delayed structural changes. Conclusions: Together, these findings demonstrate that KRAS mutational context in pancreatic ductal epithelial cells shapes early transcriptional reprogramming that actively remodels nuclear architecture and nuclear sub-compartments. This work establishes nuclear structural remodeling as a structural state of KRAS-driven epigenetic dysregulation during PDAC initiation.

## 1. Introduction

KRAS alterations represent the defining mutational event early in the etiology of ~90% of pancreatic ductal adenocarcinoma (PDAC) tumors [1]. These mutations drive extensive transcriptional and structural reprogramming, transforming normal pancreatic epithelial cells toward a preneoplastic phenotype with altered nuclear organization and signaling dynamics [2]. While KRAS has traditionally been studied for its cytoplasmic roles in activating MAPK, PI3K, and NF-κB signaling, recent work has uncovered its influence on nuclear processes, including chromatin remodeling, transcriptional regulation, and RNA processing [3,4,5]. One well-defined example is the euchromatin histone methyltransferase EHMT2, which acts downstream of KRAS G12D to form a complex with EHMT1 and WIZ. This complex deposits H3K9me2 at genomic loci that regulate cellular growth, suppress cell-cycle inhibitory networks, remodel immune, inflammatory, and injury response programs, and establish a repressive chromatin state that drives altered transcriptional output across the pancreas [6,7]. Through rewiring of epigenetic regulators such as EHMT2, KRAS signaling establishes a nuclear environment that sustains oncogenic gene expression and contributes to PDAC pathogenesis.

KRAS mutations in PDAC predominantly involve single amino acid substitutions at G12. The most commonly observed KRAS mutations in PDAC are G12D, G12V, and G12R, with additional, less frequent variants at residues such as G13 and Q61 that contribute to mutational and functional heterogeneity in the disease [8]. These mutations characterize the KRAS mutational spectrum and determine the potency and nature of downstream signaling. G12D and G12V mutations strongly activate the ERK and AKT pathways, whereas G12R exhibits reduced signaling output and is consistently associated with better patient survival across multiple large clinical cohorts [9,10,11]. Prior work has shown that KRAS G12D signaling reshapes nuclear architecture by reorganizing chromatin territories [3] and disrupting nuclear lamina integrity [12], establishing a distinct nuclear landscape that promotes gene expression programs supporting proliferation, stress tolerance, and transformation [13,14]. Moreover, components of the inner nuclear membrane have emerged as functional effectors of KRAS activity, linking oncogenic signaling to physical remodeling of nuclear structure and chromatin compaction [15]. Despite these advances, our understanding of KRAS-driven nuclear organization is based primarily on studies of KRAS G12D, leaving the extent to which other clinically prevalent KRAS variants differentially impact nuclear architecture poorly defined.

In this study, we model early mutant KRAS expression in non-cancerous pancreatic ductal epithelial cells to define the earliest molecular and structural responses to oncogenic signaling. We first profile a broad panel of PDAC-associated KRAS variants using integrated transcriptomic and phospho-proteomic approaches to establish mutation-specific regulatory programs enriched for epigenetic regulators and nuclear structural factors. Guided by the pronounced epigenetic and transcriptional heterogeneity revealed in these datasets, we then focus on the two most divergent variants, the most prevalent KRAS G12D and the clinically distinct KRAS G12R, and apply in-depth cellular imaging to examine how these distinct epigenetic states translate into differential nuclear remodeling. Together, these results delineate the earliest epigenetically driven nuclear consequences of oncogenic KRAS signaling and establish nuclear heterogeneity as a fundamental feature of variant-specific PDAC initiation.

## 2. Results

### 2.1. PDAC-Associated KRAS Signaling Initiates Early Variant-Specific Nuclear Regulatory Transcriptional Programs

**Figure 1:**
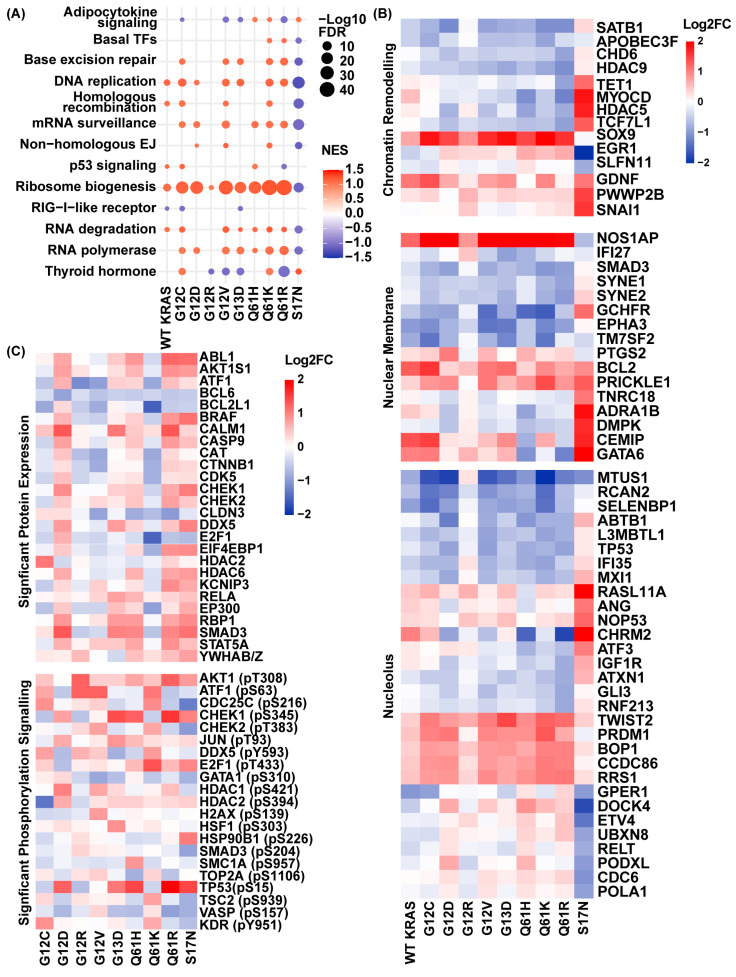
**PDAC-associated KRAS mutants drive early nuclear transcriptional and proteomic heterogeneity.** (**A**) GSEA of transcriptomes 24 h after induction of eight PDAC KRAS mutants and the dominant negative S17N control, relative to empty vector (+doxycycline), reveals selective activation of nuclear-associated transcriptional programs. (**B**) Differentially expressed genes were selected based on Gene Ontology cellular component annotations, highlighting transcriptional heterogeneity among chromatin remodelers (green), nuclear membrane-associated genes (red), and nucleolus-associated genes (blue). (**C**) Nuclear regulatory protein abundance and phosphorylation vary across KRAS variants, as measured by antibody-based profiling comparing +doxycycline samples to their matched no doxycycline (−doxy) controls.

To characterize how KRAS mutations initiate early nuclear remodeling, we performed RNA sequencing 24 h after inducing eight common PDAC-associated KRAS mutations (G12C, G12D, G12V, G12R, G13D, Q61H, Q61K, Q61R) and the dominant-negative S17N control in HPNE cells, a non-cancerous pancreatic ductal epithelial cell model. To ensure that downstream effects reflected mutational differences rather than expression levels, Western blot analysis confirmed equivalent expression of all induced KRAS variants at 24 h (Figure S1A). Transcriptomic analysis likewise showed comparable KRAS mRNA levels across conditions, with no change in NRAS or HRAS expression (Figure S1B). Subsequently, we performed gene set enrichment analysis (GSEA) using KEGG pathways, which revealed pronounced, mutation-specific activation of nuclear transcriptional programs (Figure 1A). Ribosome biogenesis, a hallmark of nucleolar remodeling, showed strong early enrichment across G12C (NES = 1.23), G12D (NES = 1.18), G12V (NES = 1.20), G13D (NES = 1.15), Q61H (NES = 1.20), Q61K (NES = 1.22), and Q61R (NES = 1.21). In contrast, G12R displayed minimal enrichment (NES = 1.02), comparable to WT KRAS (NES = 1.08), whereas S17N showed depletion (NES = −1.10). These data indicate that activation of nucleolar and ribosomal RNA programs constitutes a conserved early response to oncogenic KRAS signaling, with G12R showing activity closer to WT than to other oncogenic variants.

Beyond ribosome biogenesis, additional mutation-specific differences in pathways emerged. G12R selectively suppressed thyroid hormone signaling, whereas G12C and Q61K modestly activated this pathway, with other variants showing intermediate responses. DNA replication pathways were strongly enriched in G12C, G12D, G12V, G13D, Q61K, and Q61R, but not in WT KRAS or G12R. Base excision repair was active across most variants but notably absent in G12D, whereas non-homologous end-joining was selectively enriched in G12D, G12V, and Q61K. Additionally, homologous recombination was preferentially enriched in WT KRAS, G12C, G12D, and Q61R, highlighting variant-specific reliance on distinct DNA repair programs. The dominant-negative S17N mutant suppressed all replication and repair pathways, confirming their dependence on active KRAS signaling. RNA processing pathways also exhibited variant-specific patterns. RNA polymerase activation occurred broadly across oncogenic KRAS variants, with G12R as the notable exception. RNA degradation pathways were engaged in the presence of WT KRAS and most variants, but were absent in G12D and G12R (Figure 1A). Collectively, these data demonstrate that most PDAC-associated KRAS variants activate coordinated nucleolar, transcriptional, replication, and DNA repair programs, whereas G12R consistently exhibits lower pathway engagement, often at or below WT KRAS levels, defining a divergent early transcriptional state.

To further dissect early nuclear remodeling, we analyzed transcriptional changes in a curated set of 60 genes associated with chromatin remodeling, nuclear membrane components, and nucleolar function based on Gene Ontology cellular component annotations (Figure 1B and Table S1). Most genes showed coordinated regulation across oncogenic KRAS variants, indicating a shared early nuclear remodeling transcriptional framework. G12R again diverged, eliciting a weaker overall response, whereas S17N showed an inverse regulatory pattern compared with oncogenic KRAS mutants. This supports a model in which the attenuated downstream signaling associated with G12R preserves nuclear regulatory states that other oncogenic variants actively remodel. For example, among chromatin remodelers, *HDAC5*, a key negative regulator of transcription, was downregulated across most KRAS mutants with a log2 fold change (FC) of −0.73 in G12D, −0.87 in G12V, −0.90 in Q61H, −0.49 in Q61K, and -0.81 in Q61R relative to empty vector (+doxycycline). In contrast, WT KRAS (+0.17) and G12R (+0.16) maintained near-baseline *HDAC5* expression, and S17N showed strong upregulation (+1.73). Similarly, *SOX9*, encoding a chromatin-associated transcription factor implicated in lineage specification and chromatin remodeling, was robustly induced across oncogenic KRAS variants (range +1.44 to +2.13), whereas WT KRAS and G12R showed more modest activation (+0.74 and +0.85, respectively) and S17N showed no induction (−0.05). *SATB1*, a global chromatin organizer, followed a similar pattern, with substantial downregulation in G12D (−1.13), G12V (−0.88), G13D (−0.80), Q61K (−1.10), and Q61R (−1.09). WT KRAS showed only a modest reduction (−0.43), G12R exhibited minimal change (−0.05), and S17N demonstrated increased expression (+0.32). Genes associated with nuclear structure and nucleolar function showed analogous trends. *NOS1AP*, a nucleoporin-associated gene linked to nuclear envelope organization, was strongly induced across most PDAC-associated KRAS mutants (G12C: +2.06, G12D: +2.06, G12V: +2.11, G13D: +2.01, Q61H: +2.19, Q61K: +2.61, Q61R: +1.90), reflecting coordinated remodeling of nuclear envelope dynamics, whereas G12R showed attenuated induction (+0.84) similar to WT KRAS (+1.21) while S17N repressed expression (−0.66). The nucleolar gene *CCD86* showed modest upregulation across most variants (G12C: +0.85, G12D: +0.89, G12V: +0.82, Q61K: +1.03, Q61R: +0.99), with a comparatively weaker induction in WT KRAS (+0.43) and G12R (+0.30) that closely resembled the S17N profile (+0.10). Similarly, *ABTB1*, which encodes a zinc-finger nucleolar protein, was consistently repressed among most oncogenic KRAS variants (G12C: −0.37, G12D: −1.24, G12V: −0.80, Q61H: −1.00, Q61K: −0.78, Q61R: −0.78), with negligible change in WT KRAS (−0.04) and G12R (+0.03) and increased expression in the dominant-negative S17N control (+0.58) (Figure 1B).

To quantify overall remodeling, we calculated the Nuclear Transcriptional Remodeling Score (NTRS) as the mean log2 FC in upregulated and downregulated nuclear-associated genes for each condition (Figure S1C). G12D, G12V, G13D, Q61H, and Q61R showed comparable positive NTRS values (0.59 to 0.65) together with similarly strong negative scores (−0.52 to −0.72), consistent with coordinated bidirectional transcriptional reprogramming. G12C and Q61K exhibited the highest positive NTRS values (0.72 and 0.73, respectively), alongside pronounced negative scores, indicating robust remodeling capacity. WT KRAS demonstrated more modest bidirectional activity (positive NTRS 0.48; negative −0.37), lower than that of the oncogenic variants, consistent with a comparatively restrained transcriptional remodeling profile. G12R displayed further attenuation, with a reduced positive NTRS of 0.35 and a substantially weaker negative score of −0.19, reflecting even more limited nuclear transcriptional remodeling than WT KRAS. The dominant-negative S17N control produced the highest bidirectional NTRS values. However, as seen in Figure 1B, these results reflect an inverted transcriptional program relative to oncogenic KRAS variants rather than enhanced activation of the typical nuclear transcriptional response. Together, this focused analysis reveals that most oncogenic KRAS variants drive coordinated early transcriptional remodeling of chromatin, nuclear envelope, and nucleolar regulators, whereas G12R remains comparatively muted, with activity at or below WT KRAS levels.

### 2.2. Phospho-Proteomic Profiling Reveals Divergent Nuclear Signaling Downstream of Oncogenic KRAS

Next, we performed phospho-proteomic profiling using an antibody-based array to quantify changes in protein abundance and phosphorylation across KRAS variants, comparing +doxycycline samples to their matched no doxycycline (−doxy) controls. To assess how variant-specific KRAS signaling is reflected at the level of nuclear regulatory proteins, we examined representative chromatin remodelers, RNA-binding factors, and transcriptional effectors. Analysis of nuclear-associated proteins revealed substantial heterogeneity in early protein abundance across KRAS variants. G12D and Q61R, together with S17N, exhibited the highest overall protein abundance following induction. G13D and Q61H also increased protein abundance, though to a lesser extent. By comparison, G12C, G12V, and Q61K displayed minimal induction or reduced abundance relative to their uninduced controls, underscoring divergence in early nuclear signaling outputs. DDX5, a DEAD-box RNA helicase central to RNA processing and nuclear regulation, displayed the strongest induction in G12D relative to pre-induction (−doxycycline) controls (+0.81 log2FC), followed by G13D (+0.79), Q61R (+0.48), and Q61H (+0.35). In contrast, G12C, G12V, and Q61K exhibited modest reductions (−0.29, −0.22, and −0.21, respectively), and G12R remained essentially unchanged (+0.01). The dominant-negative S17N displayed marked induction (+0.99). HDAC6 followed a similar pattern, with elevated abundance in G12D (+0.68) and Q61H (+0.66), modest increases in Q61R (+0.22), minimal change in G12R (+0.11) and Q61K (+0.08), and reduced abundance in G12C (−0.04), G12V (−0.25), and G13D (−0.17). Smad3, a TGF-β effector with nuclear roles in chromatin organization and transcriptional regulation, showed the most pronounced G12R-specific divergence. Smad3 abundance increased strongly in G12D (+1.34), G13D (+1.01), Q61H (+0.95), and Q61R (+0.98), with modest induction in G12C (+0.23) and a reduction in G12R (−0.50) (Figure 1C). At the level of total protein abundance, these examples indicate that most oncogenic KRAS variants reinforce early nuclear RNA and chromatin regulatory capacity, while G12R shows limited engagement of these responses and divergence at specific nuclear effectors.

Building on these differences in protein abundance, we next examined phosphorylation to determine whether post-translational regulation of nuclear factors varied across KRAS mutants. HDAC1 phosphorylation at Ser421, a modification required for enzymatic activity and transcriptional repression [1], displayed clear variant specificity (Figure 1C). G12D showed the strongest induction (+1.03), followed by G12V (+0.73) and Q61R (+0.52), whereas G13D, Q61K, G12C, and Q61H exhibited minimal to modest changes (−0.08 to +0.32). G12R showed reduced phosphorylation (−0.15), closely resembling S17N (−0.37), indicating attenuated activation of this epigenetic regulator. A comparable pattern was found for HDAC2 phosphorylation at Ser394, which facilitates enzymatic activation, heterodimerization with HDAC1, and recruitment to transcriptionally active chromatin [2]. HDAC2 Ser394 phosphorylation was highest in G12D (+0.68) and broadly elevated across G13D (+0.41), Q61H (+0.40), Q61R (+0.32), G12V (+0.25), and Q61K (+0.25) (Figure 1C). In contrast, phosphorylation at this site was reduced in G12C (−1.33) and G12R (−0.36). Phosphorylation of DDX5 at Tyr593, a modification that regulates nuclear export and signaling activity [3], showed a distinct variant-specific distribution (Figure 1C). Q61K (+0.80), G12C (+0.78), and G13D (+0.78) exhibited the highest levels of Tyr593 phosphorylation, with intermediate increases in G12R (+0.65), G12V (+0.46) and Q61H (+0.41). Q61R showed a negligible change at this site (−0.03). Notably, G12D demonstrated reduced Tyr593 phosphorylation (−0.63), distinguishing it from other oncogenic KRAS variants. Lastly, phosphorylation of TP53 at Ser15, a canonical DNA damage response modification mediated by ATM and ATR kinases [4], further distinguished mutant-specific nuclear stress signaling (Figure 1C). The strongest induction of p53 Ser15 phosphorylation was found with Q61R (+2.39), with substantial increases also detected in Q61H (+1.45), G12D (+1.26), and G13D (+1.08). G12V and Q61R showed minimal changes at this site (+0.05 and +0.18, respectively), while G12C (−0.45) and Q61K (−0.39) demonstrated reduced phosphorylation. G12R showed the greatest repression (−0.62), consistent with diminished early DNA damage signaling and nuclear oncogenic stress relative to other KRAS mutants (Figure 1C). These phosphorylation patterns indicate that oncogenic KRAS variants drive early nuclear regulation not only through changes in protein abundance, but also via variant-specific post-translational signaling. Across chromatin regulators, RNA-binding proteins, and DNA damage response effectors, most PDAC-associated KRAS mutants engage coordinated phosphorylation programs that reinforce nuclear regulatory activity within 24 h of activation. G12R consistently shows attenuated or divergent phosphorylation across multiple regulatory nodes, often resembling the dominant-negative control.

When integrated with the transcriptional and total protein abundance data, these results reveal that oncogenic KRAS mutations encode a spectrum of early nuclear regulatory states in non-cancerous pancreatic epithelial cells within 24 h, thereby priming the nucleus for progressive dysregulation as mutant KRAS expression persists. G12D and G12R occupy opposing ends of this spectrum, with G12D exhibiting broad engagement of nuclear regulatory and stress-response pathways, and G12R displaying attenuated or divergent signaling across transcriptional, proteomic, and phospho-proteomic layers. Intermediate variants such as G12C and Q61K show pathway-specific deviations within this continuum. Together, these findings support a model in which early mutant KRAS signaling establishes variant-specific nuclear trajectories that likely influence subsequent chromatin remodeling and nuclear architecture reorganization during PDAC initiation.

### 2.3. KRAS G12D and G12R Drive Distinct Nuclear and Subnuclear Remodeling in Pancreatic Epithelial Cells

**Figure 2:**
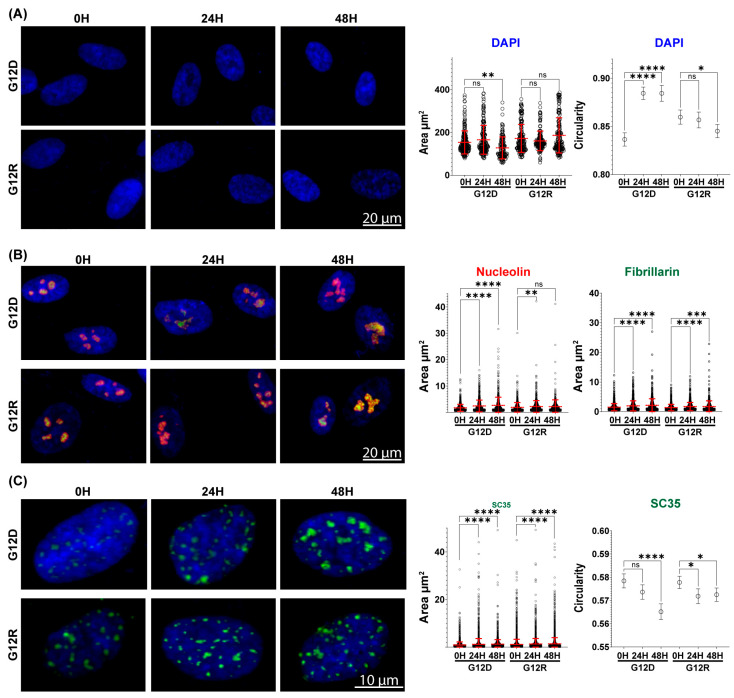
**KRAS G12D and G12R differentially remodel nuclear and subnuclear architecture in non-cancerous HPNE cells.** (**A**) Nuclear area and circularity were quantified at 24 and 48 h following induction of KRAS G12D or G12R, revealing variant-dependent changes in nuclear size and shape, as detected by DAPI staining. Cells analyzed for nuclear quantification were: G12D 0 h (*n* = 290), 24 h (*n* = 222), 48 h (*n* = 168); G12R 0 h (*n* = 290), 24 h (*n* = 262), 48 h (*n* = 290). (**B**) Nucleolar organization was assessed by quantifying nucleolar area using nucleolin (red) and fibrillarin (green) across conditions. Cells analyzed were: G12D 0 h (*n* = 442), 24 h (*n* = 401), 48 h (*n* = 312); G12R 0 h (*n* = 270), 24 h (*n* = 278), 48 h (*n* = 207). (**C**) Spliceosomal organization was evaluated by measuring the area and circularity of individual spliceosomal puncta using the SC35 marker (green), revealing mutation-dependent changes in spliceosomal compartment structure. Cells analyzed were: G12D 0 h (*n* = 275), 24 h (*n* = 268), 48 h (*n* = 251); G12R 0 h (*n* = 309), 24 h (*n* = 198), 48 h (*n* = 229). n.s., not significant; * *p* < 0.05, ** *p* < 0.01, *** *p* < 0.001, **** *p* < 0.0001; Data presented as mean ± SD (red error bars) or mean ± 95% confidence interval (black error bars). All conditions were performed in biological triplicate.

The preceding multi-omics analyses revealed substantial heterogeneity in early nuclear regulatory signaling across PDAC-associated KRAS variants, with KRAS G12D and G12R representing opposing extremes of transcriptional and proteomic remodeling. To determine whether these molecular differences translate into measurable changes in nuclear organization, we focused quantitative imaging analyses on G12D and G12R. Nuclear features were assessed at 24 and 48 h following KRAS induction to allow sufficient time for transcriptional changes to manifest morphologically. KRAS G12R expression did not significantly alter mean nuclear area at either 24 or 48 h relative to the 0 h control (Figure 2A). G12D produced no change at 24 h but resulted in a 16% reduction in nuclear area by 48 h, indicating nuclear compaction over time. Nuclear shape was then assessed by circularity, where a value of 1 represents a maximally circular nucleus. G12D-expressing nuclei increased in circularity from 0.84 at baseline to 0.88 by 24 h, which was maintained at 48 h, consistent with progressive nuclear rounding accompanying compaction. By comparison, G12R expression showed no change at 24 h and only a limited decrease at 48 h (0.86 to 0.84) (Figure 2A). These measurements indicate that G12D rapidly remodels nuclear architecture, whereas G12R induces minimal and delayed changes.

Given the enrichment of ribosome biogenesis pathways (Figure 1A) and nucleolar-associated transcriptional programs (Figure 1B), we next examined whether mutant KRAS alters nucleolar organization. Nucleolar size was quantified using nucleolin and fibrillarin to capture changes across functionally distinct nucleolar sub-compartments. Fibrillarin is enriched within the fibrillar center and at the fibrillar center–dense fibrillar component interface, while nucleolin localizes predominantly to the dense fibrillar and granular components and is often enriched at the dense fibrillar–granular component interface, enabling assessment of coordinated nucleolar remodeling. At baseline, G12D-expressing cells displayed comparable areas for individual nucleolar components, with nucleolin- and fibrillarin-positive areas averaging 1.72 ± 1.32 µm^2^ and 1.51 ± 1.21 µm^2^, respectively. Following 24 h of G12D induction, the nucleolin-positive area increased by 41% to 2.43 µm^2^ and expanded further to 2.65 µm^2^ by 48 h (54%). Fibrillarin-positive regions showed a similar trajectory, increasing to 1.97 µm^2^ (30%) at 24 h and 2.14 µm^2^ (41%) at 48 h. In contrast, G12R-expressing cells had similar baseline values (1.88 ± 1.82 µm^2^ for nucleolin and 1.38 ± 1.09 µm^2^ for fibrillarin) but showed limited expansion over time. After 24 h, nucleolin and fibrillarin areas increased by 16% (2.19 µm^2^) and 25% (1.73 µm^2^), respectively, and these values were largely retained at 48 h (Figure 2B). Thus, G12D drives coordinated enlargement of nucleolar sub-compartments, consistent with elevated ribosome biogenesis, whereas G12R supports only limited nucleolar expansion.

Finally, we examined spliceosomal organization using SC-35 to quantify compartment area and circularity. In KRAS G12D-expressing HPNE cells, the mean spliceosomal area increased by 31% after 24 h and remained elevated at 48 h, reaching a total increase of 39% (Figure 2C). G12R expression elicited a delayed and attenuated response, with only a 16% increase at 24 h and a total increase of 21% at 48 h. Analysis of SC-35 circularity revealed subtle but reproducible reorganization in spliceosomal architecture over time. Circularity decreased progressively following G12D induction, from a baseline mean of 0.58 to 0.57 at 24 h and 0.56 at 48 h (Figure 2C), indicating an increase in structural complexity within nuclear speckles. By comparison, G12R induction was associated with a smaller reduction in circularity, decreasing from 0.58 at baseline to 0.57 at both 24 and 48 h without further progression. These measurements show that at 48 h, G12D drives an additional 18% expansion of spliceosomal compartments compared with G12R, supporting more robust and sustained remodeling of spliceosomal organization. Overall, quantitative imaging indicates that KRAS mutations differentially influence early nuclear organization. KRAS G12D exhibits pronounced changes in nuclear size, shape, and subnuclear compartment structure, whereas KRAS G12R shows more limited and delayed remodeling across these features. These findings establish variant-specific differences in nuclear organization and prompted further examination of nuclear architecture at greater spatial resolution.

### 2.4. KRAS G12D and G12R Differentially Shape Volumetric Nuclear Architecture

**Figure 3:**
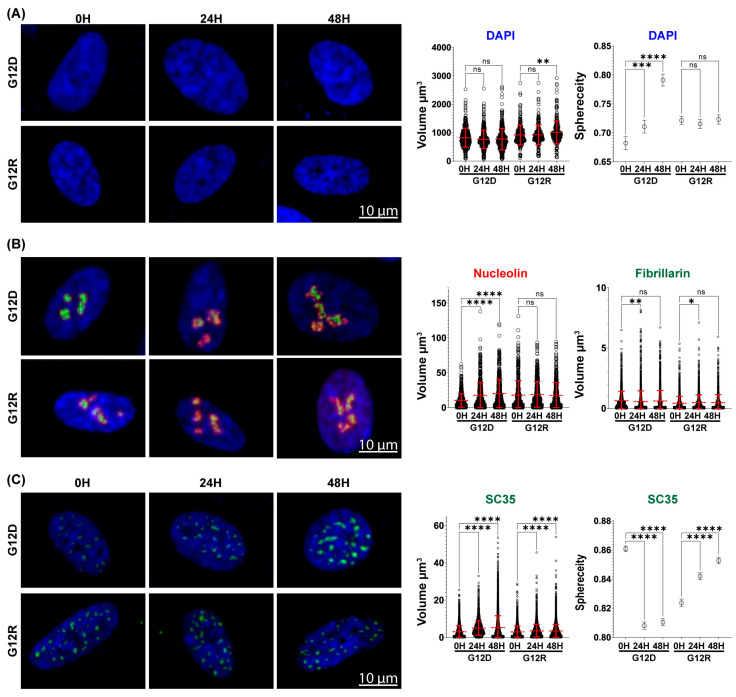
**KRAS G12D and G12R differentially exhibit distinct patterns of nuclear and subnuclear organization across nuclear volume.** Nuclear and subnuclear structures were reconstructed across the full nuclear volume in non-cancerous HPNE cells expressing KRAS G12D or KRAS G12R at 0, 24, and 48 h. (**A**) Nuclear volume and sphericity were quantified from DAPI staining to assess differences in nuclear geometry associated with G12D and G12R expression. Cells analyzed for nuclear quantification were: G12D 0 h (*n* = 401), 24 h (*n* = 414), 48 h (*n* = 460); G12R 0 h (*n* = 292), 24 h (*n* = 287), 48 h (*n* = 278). (**B**) Nucleolar organization was evaluated by measuring the volume of nucleolin-positive regions (red) and fibrillarin-positive regions (green), capturing changes across nucleolar sub-compartments. Cells analyzed were: G12D 0 h (*n* = 237), 24 h (*n* = 254), 48 h (*n* = 187); G12R 0 h (*n* = 167), 24 h (*n* = 159), 48 h (*n* = 162). (**C**) Spliceosomal compartment organization was quantified by measuring the volume and sphericity of SC-35-positive speckles (green), revealing differential effects of G12D and G12R on spliceosomal architecture. Cells analyzed were: G12D 0 h (*n* = 201), 24 h (*n* = 221), 48 h (*n* = 243); G12R 0 h (*n* = 172), 24 h (*n* = 146), 48 h (*n* = 187). n.s., not significant; * *p* < 0.05, ** *p* < 0.01, *** *p* < 0.001, **** *p* < 0.0001; Data presented as mean ± SD (red error bars) or mean ± 95% confidence interval (black error bars). All conditions were performed in biological triplicate.

To extend our nuclear analyses beyond planar measurements, we employed high-resolution spinning disc confocal microscopy to generate depth-resolved volumetric reconstructions of nuclei and subnuclear compartments. This approach enabled assessment of nuclear remodeling across the full nuclear volume and allowed direct comparison with patterns identified by 2D imaging. In KRAS G12D-expressing HPNE cells, mean nuclear volume measured 823.0 µm^3^ at baseline and decreased modestly to 762.2 µm^3^ (−7.3%) after 24 h and 773.3 µm^3^ (−6.0%) after 48 h. Although these changes did not reach statistical significance, the trend toward volumetric compaction parallels the reduction in nuclear area detected by 2D analysis, supporting consistent nuclear condensation following G12D induction. Relative to G12D, KRAS G12R-expressing cells exhibited larger nuclear volumes at baseline (917.9 µm^3^), remained stable at 24 h (924.0 µm^3^), and increased significantly to 1012.0 µm^3^ (+10.2%) after 48 h. To further characterize nuclear shape across the nuclear volume, we quantified nuclear sphericity as a 3D analog of circularity. Nuclear sphericity increased progressively following G12D induction, rising from 0.68 at baseline to 0.71 at 24 h and further to 0.79 at 48 h, indicating progressive nuclear rounding. G12R expression did not significantly alter sphericity, which remained near baseline across all time points (0.72 at baseline, 0.71 at 24 h, and 0.72 at 48 h) (Figure 3A). Thus, nuclear sphericity therefore diverges between G12D and G12R, reinforcing variant-specific differences.

Volumetric analysis was then extended to nucleolar compartments to determine whether KRAS-driven changes in nuclear organization were accompanied by alterations in subnuclear architecture. Nucleolar volume was quantified using nucleolin and fibrillarin to assess distinct nucleolar regions across the full nuclear volume. In KRAS G12D-expressing cells, nucleolin-defined nucleolar volume increased substantially, rising by 71% after 24 h and reaching a 95% increase by 48 h relative to baseline (Figure 3B). Over the same time frame, fibrillarin-defined volume decreased by 11% at 24 h, returning to baseline by 48 h. A different pattern emerged in G12R-expressing cells. Nucleolin volume remained unchanged at both 24 h and 48 h, whereas fibrillarin volume increased modestly by 14% at 24 h and returned to baseline by 48 h (Figure 3B). Consistent with the 2D results, volumetric analysis confirms nucleolar expansion following G12D induction and further reveals compartment-specific reorganization rather than uniform enlargement. G12R, in comparison, exhibits only modest and transient nucleolar remodeling.

Given the pronounced nucleolar reorganization identified downstream of KRAS G12D, we next determined whether changes in nuclear architecture extended to additional RNA-associated nuclear compartments. To address this, we examined the volumetric organization of the spliceosomal compartment using the SC-35 marker across the full nuclear volume. Following KRAS G12D induction, SC-35 volume increased significantly, rising by 60% at 24 h and further to 71% at 48 h relative to baseline (Figure 3C). This expansion was accompanied by a decrease in sphericity, from 0.86 at baseline to 0.80 at 24 h and 0.81 at 48 h, reflecting altered spliceosomal speckle organization. G12R expression produced a more restrained response. SC-35 volume increased modestly, by 17% at 24 h and by 12% at 48 h, while spliceosomal sphericity increased slightly from 0.82 at baseline to 0.84 at 24 h and 0.85 at 48 h (Figure 3C). Thus, KRAS G12D drives an additional 59% expansion of the spliceosomal compartment relative to G12R, accompanied by a decrease in sphericity that reflects altered speckle geometry. Overall, volumetric imaging refines the differences identified by 2D analysis by revealing how variant-specific nuclear changes are organized across the nuclear volume. KRAS G12D is associated with coordinated alterations in nuclear geometry, compartment-specific nucleolar reorganization, and sustained expansion of spliceosomal structures, whereas KRAS G12R maintains relatively stable architecture with only limited subnuclear adjustment. These depth-resolved measurements demonstrate that KRAS mutational context influences not only the magnitude but also the spatial organization of early nuclear remodeling, in a manner consistent with the transcriptional and proteomic programs defined earlier.

### 2.5. Macromolecular Architecture Reveals Mutation-Specific Reorganization of Nuclear Sub-Compartments

To examine nuclear remodeling at higher spatial resolution, we used three-dimensional super-resolution microscopy to visualize the macromolecular organization of nuclear sub-compartments following KRAS activation. Single-cell STED imaging was performed at 24 and 48 h, focusing on representative nuclei selected based on the nucleolar phenotypes identified in the preceding volumetric analyses for each KRAS variant. At 0 h, both G12D and G12R nuclei show several rounded, smoothly contoured nucleolin-positive domains with clearly separated fibrillarin puncta within each compartment, consistent with organized nucleolar subcompartments. By 24 h, both mutants display nucleolin structures that are slightly enlarged and more irregular, and in G12D, the fibrillarin signal looks less crisp and begins to cluster within the brightest nucleolin regions, whereas in G12R, the puncta aggregate but remain smaller and more distinctly separated. By 48 h, G12D nucleoli are clearly more elongated and expanded, with fibrillarin signal spread broadly throughout much of each nucleolin-defined compartment, while G12R nucleoli stay comparatively rounded with limited elongation and fibrillarin still concentrated in more compact foci within nucleolin-dense zones, reflecting more constrained nucleolar remodeling over time.

**Figure 4:**
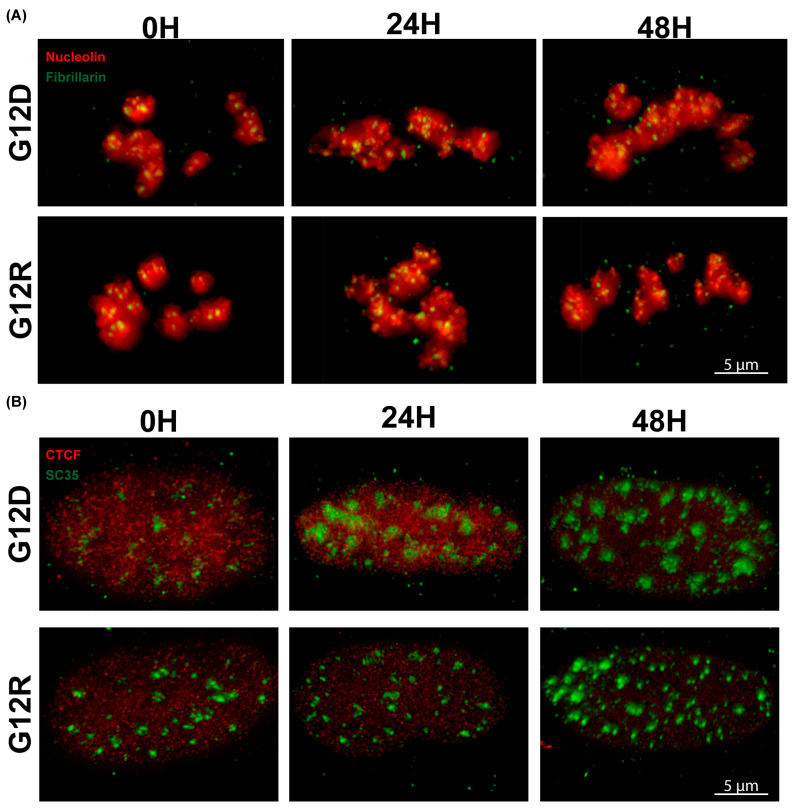
**KRAS mutant-dependent reorganization of nucleolar and spliceosomal macromolecular architecture.** Nucleolar and spliceosomal organization was examined in non-cancerous HPNE cells expressing KRAS G12D or KRAS G12R at 0, 24, and 48 h. Nanoscale organization was visualized using single-cell STED microscopy. (**A**) Nucleolin (red) and fibrillarin (green) localization defined nucleolar architecture over time. KRAS G12D expression was associated with reduced compaction and increased spatial dispersion of the fibrillarin-defined compartment, whereas KRAS G12R produced more limited changes relative to baseline. (**B**) Spliceosomal architecture was evaluated using SC-35 (green) with CTCF (red) delineating the nuclear boundary. At baseline, SC-35 formed a compact core structure. During KRAS G12D expression, additional SC-35 signal accumulated around this core, resulting in pronounced expansion and reorganization of the spliceosomal compartment. KRAS G12R showed weaker and delayed SC-35 accumulation, leading to comparatively modest structural changes.

Spliceosomal organization displayed similarly mutation-dependent differences. At baseline, SC-35-positive speckles were small and sparsely distributed throughout the nucleoplasm (Figure 4B). Following G12D induction, both speckle number and volume increased by 24 h, forming larger assemblies distributed across the nucleus. By 48 h, these structures appeared as densely packed clusters composed of multiple adjacent foci, indicating extensive reorganization of active splicing domains. This pattern is consistent with cooperative clustering of multiple spliceosomal subunits rather than uniform enlargement of individual speckles. Under G12R expression, spliceosomal architecture remained relatively stable at 24 h, with modest expansion of individual SC-35-positive foci evident at 48 h (Figure 4B), suggesting limited reorganization relative to G12D.

At nanoscale resolution, super-resolution imaging places the transcriptional, proteomic, and volumetric differences identified earlier into a coherent architectural framework. Structural features such as nucleolin fragmentation, spatial dispersion of fibrillarin, and clustering of SC-35-positive speckles exemplify a coordinated nuclear reorganization program associated with KRAS G12D activation. In contrast, preservation of compact nucleolar organization and limited spliceosomal rearrangement under KRAS G12R reflects a more stable nuclear state. Together, these convergent structural signatures indicate that KRAS mutational context is translated into distinct nuclear organizational states early after activation, linking variant-specific signaling to the spatial patterning of nuclear compartments during early pancreatic epithelial transformation.

## 3. Discussion

This study defines how heterogeneity among oncogenic KRAS mutations drives distinct nuclear remodeling programs during the earliest stages of pancreatic tumorigenesis. Although KRAS mutations occur in more than 90% of PDAC, the mechanisms by which individual mutant KRAS proteins differentially influence nuclear structure and organization have remained unclear. Our data identify KRAS G12D and G12R as functionally divergent mutations, motivating focused analysis of their nuclear effects in inducible, non-transformed HPNE models that capture a preneoplastic cellular context. Our previous work demonstrated that KRAS G12D rapidly reshapes chromatin territories within 24 h, driving early nuclear reprogramming through the redistribution of H3K27me3- and H3K9me3-marked heterochromatin and the reorganization of super-enhancer domains [5]. Here, we extend these findings by linking early epigenetic remodeling directly to changes in nuclear architecture across multiple spatial scales, integrating transcriptional, proteomic, and imaging-based analyses. Our results suggest that early oncogenic KRAS signaling establishes mutation-specific nuclear organizational states that parallel differences in transcriptional engagement and biosynthetic demand. Rather than arising as a late consequence of transformation, nuclear remodeling occurs rapidly and in coordination with transcriptional reprogramming, suggesting that nuclear organization adapts alongside gene expression programs during the earliest phases of KRAS-driven oncogenesis. In this framework, nuclear architecture reflects the magnitude and coordination of oncogenic signaling rather than a uniform downstream response.

In cells expressing KRAS G12D, early activation of nucleolar, DNA replication, and RNA processing programs likely reflects a downstream transcriptional consequence of heterochromatin redistribution and super-enhancer reorganization. Consistent with this model, G12D is associated with early changes in nuclear geometry followed by progressive nuclear compaction, aligning with prior studies showing that KRAS-driven ERK signaling regulates nuclear size and mechanics through Emerin [6]. In contrast, KRAS G12R elicits a markedly attenuated nuclear response, with limited changes in nuclear shape and subnuclear organization. This comparison extends this paradigm in two critical ways. First, we show that substitutions at the same KRAS codon produce variant-specific differences in nuclear organization. Second, we demonstrate that these differences go beyond nuclear envelope dynamics to include nucleolar and spliceosomal compartments, revealing subnuclear structures as previously unrecognized targets of KRAS-driven transcriptional and architectural heterogeneity. Notably, these divergent nuclear states emerge within 24 h of mutant KRAS expression in pancreatic epithelial cells, highlighting mutation-specific differences in KRAS signaling output and effector engagement, which have been shown to vary substantially across PDAC-associated variants [7], as a key determinant of early nuclear organization.

To further understand how KRAS reshapes the nuclear landscape, we focused on the nucleolus, the largest and most metabolically active nuclear sub-compartment [8]. Oncogenic RAS-ERK1/2 signaling has been shown to promote nucleolin phosphorylation, thereby enhancing its rRNA-binding activity and stimulating ribosome biogenesis in PDAC cells [9]. In line with this mechanism, we show that KRAS G12D is associated with strong transcriptional enrichment of ribosome biogenesis pathways, accompanied by pronounced enlargement of nucleolin- and fibrillarin-defined nucleolar compartments, consistent with increased ribosomal biosynthetic capacity. By contrast, KRAS G12R shows substantially weaker enrichment of ribosome biogenesis programs and only modest nucleolar reconfiguration, reinforcing prior observations that this variant engages downstream signaling pathways less efficiently and in a more heterogeneous manner [10,11,12]. Nucleolar hypertrophy is a hallmark of cancer cells and drives increased ribosome biogenesis, translational output, and proliferative potential [13]. G12R cells show no nucleolin expansion, indicating that these mutants maintain a fundamentally lower biosynthetic capacity than G12D cells. This constrained ribosomal output may impose limits on proliferative capacity, potentially contributing to the reduced aggressiveness associated with G12R-driven tumors.

Divergent patterns were also evident within RNA processing compartments. Nuclear speckles actively regulate gene expression, and proximity to speckles correlates with transcriptional output [14]. Oncogenic KRAS alters splicing factor phosphorylation and drives widespread changes in alternative splicing in lung cancer, with effects that depend on specific mutations such as G12V and Q61H [15]. Consistent with this variant-specific regulation, KRAS G12D reorganized SC-35-marked spliceosomal compartments, reflecting heightened engagement of the splicing machinery. By contrast, KRAS G12R preserved spliceosomal architecture, indicating that reduced transcriptional activation is accompanied by stable RNA processing organization. The attenuated SC-35 expansion in G12R cells likely reflects lower engagement of splicing-associated transcriptional programs, with potential consequences for alternative splicing patterns that shape tumor biology.

Thus, comparison of KRAS G12D and KRAS G12R provides deeper mechanistic insight into how mutation-specific KRAS signaling shapes early nuclear behavior. Although both variants engage canonical RAS pathways, KRAS G12D is associated with more rapid and coordinated reorganization of nuclear, nucleolar, and spliceosomal compartments, whereas KRAS G12R exhibits delayed and attenuated changes across these features. Phospho-proteomic profiling supports this distinction, revealing stronger engagement of epigenetic regulators and DNA damage-associated nuclear signaling downstream of KRAS G12D, consistent with elevated nuclear stress accompanying high-output oncogenic signaling. The accompanying nuclear and subnuclear reorganization, therefore, likely reflects an adaptive response to increased transcriptional and biosynthetic demand, rather than isolated downstream effects. In this context, the more restrained nuclear configuration associated with KRAS G12R suggests preservation of a comparatively homeostatic nuclear state despite oncogenic activation. Notably, this nuclear phenotype aligns with prior clinical observations linking KRAS G12R to more favorable outcomes in PDAC [16], raising the possibility that early differences in nuclear organization reflect broader distinctions in oncogenic burden imposed by specific KRAS variants.

These mutation-specific nuclear states may also have implications for therapeutic vulnerability. The strong engagement of ribosome biogenesis and RNA processing machinery associated with KRAS G12D suggests a greater reliance on biosynthetic and transcriptional capacity, potentially sensitizing these cells to perturbations of nucleolar function or RNA processing pathways. Conversely, the attenuated nuclear remodeling observed under KRAS G12R expression may confer relative resistance to such interventions, consistent with its weaker and more heterogeneous signaling output. More broadly, these findings raise the possibility that KRAS mutation subtype could influence responses not only to therapies targeting downstream biosynthetic pathways, but also to emerging pan-KRAS or pathway-agnostic inhibitors that aim to suppress oncogenic KRAS signaling across variants. In this context, nuclear organization may serve as an early integrative readout of how effectively such therapies disrupt the transcriptional and biosynthetic programs sustained by distinct KRAS mutations and thus represents an important area for further functional research.

Together, these findings identify nuclear organization as an early and quantifiable feature associated with oncogenic KRAS signaling rather than merely a passive byproduct of transformation. Distinct KRAS mutations correspond to different nuclear architectural states during early pancreatic tumorigenesis, suggesting that KRAS variant identity may influence nuclear remodeling trajectories. Importantly, the present study defines structural nuclear states, and additional investigations will be required to determine how these architectural differences translate into functional consequences for disease progression. As with any in vitro system, the HPNE model reflects a defined biological context. These cells express HPV16 E6/E7, resulting in TP53 and RB inactivation, pathways that regulate chromatin organization, DNA damage signaling, nucleolar dynamics, and cell cycle control [17]. Inactivation of TP53 and RB, together with sustained proliferation and standard culture conditions, likely contributes to baseline nuclear architectural features distinct from primary pancreatic epithelial cultures. Although this background may modulate KRAS-driven responses, the relative differences among KRAS variants remain informative within the same controlled context. Inclusion of WT KRAS and the dominant-negative S17N control establishes a signaling baseline and supports that the graded architectural and transcriptional differences across oncogenic mutants reflect KRAS-dependent signaling rather than a uniform E6/E7 effect. Future studies in TP53- and RB-intact systems, organoids, and patient-derived models will be important for assessing generalizability.

Nuclear features, such as nucleolar volume and SC35 speckle organization, are influenced by cell cycle stage [18,19], which is an additional consideration when interpreting architectural phenotypes. While the present study focused on defining structural states across KRAS variants within a consistent experimental framework, future studies incorporating cell cycle profiling would further refine the interpretation of how proliferation dynamics intersect with KRAS-driven remodeling. Similarly, complementary measurements of rRNA synthesis, translational output, and stress responses could help determine how these structural states relate to cellular function. The volumetric and morphometric analyses presented capture robust population-level trends in nuclear and subnuclear organization. Super-resolution STED imaging further provided nanoscale architectural insight into nucleolar and spliceosomal organization, complementing the quantitative patterns detected by 2D and 3D imaging approaches. Although single-cell variability at finer scales warrants additional investigation, the combined imaging strategies establish a reproducible structural framework for comparing KRAS variants. In addition, the 24 to 48 h window examined in this study was designed to capture early nuclear remodeling events following oncogenic KRAS activation and may not reflect longer-term or steady-state configurations. Determining the persistence and reversibility of these nuclear states, particularly in the context of KRAS pathway inhibition, will be important for evaluating therapeutic relevance. Although the architectural patterns align with known clinical differences among KRAS variants, direct evaluation of how these nuclear features relate to patient outcomes will benefit from future clinicopathological studies incorporating nuclear morphometry in molecularly annotated PDAC specimens.

In summary, this study provides the first systematic and quantitative comparison of how PDAC-associated KRAS variants differentially sculpt nuclear architecture during early oncogenic signaling responses. By defining nuclear organization as a measurable structural correlate of oncogenic genotype, this framework advances our understanding of KRAS-driven heterogeneity and establishes a foundation for further functional studies that may inform the development of therapies tailored to specific KRAS variants in PDAC.

## 4. Materials and Methods

### 4.1. Generation of Plasmids and Lentivirus

The wild-type KRAS gene (NM_004985) was inserted into the pTMONRB-TRE-MCS-I2-TagRFP-EFS-rtTA-2A-Blast using In-Fusion cloning (Takara Bio, Shiga, Japan). PDAC-relevant KRAS mutations (G12C, G12D, G12V, G12R, G13D, Q61H, Q61K, Q61R) and the dominant-negative mutant S17N were generated using the QuikChange II XL Site-Directed Mutagenesis Kit (Agilent, La Jolla, CA, USA). Plasmids were subsequently packaged at the Versiti Blood Research Institute and Medical College of Wisconsin Viral Vector Core.

### 4.2. Generation and Maintenance of Inducible KRAS Mutant HPNE Cell Lines

To establish inducible KRAS mutant pancreatic ductal epithelial models, hTERT-immortalized Human Pancreatic Nestin-Expressing cells transformed with HPV-16 E6/E7 (hTERT-HPNE E6/E7; ATCC, CRL-4036) were used as the parental line. Cells were cultured and maintained under standard conditions in a defined growth medium (75% glucose-free DMEM, 25% Medium M3 Base) supplemented with L-glutamine (2 mM), sodium bicarbonate (1.5 g/L), fetal bovine serum (5%), human recombinant EGF (10 ng/mL), D-glucose (5.5 mM), and puromycin (750 ng/mL). Cells were transduced with lentiviral particles encoding KRAS variants at a multiplicity of infection (MOI) of 10 with LentiTrans added at 1× concentration to enhance transduction efficiency. Following transduction, cells were cultured for 72 h before selection with blasticidin (10 µg/mL). Blasticidin-resistant clones were isolated and expanded to confluency. Inducible expression of KRAS variants was achieved by treatment with doxycycline (1 µg/mL) for 24 or 48 h prior to downstream analyses.

### 4.3. RNA-Seq Analysis

Cells were plated at approximately 2 × 10^6^ cells/dish in 10 cm^2^ dishes and induced to express KRAS with doxycycline the following morning. Twenty-four hours after adding doxycycline, total RNA was extracted from cell lysates using the Qiazol RNA extraction method (Qiagen, Hilden, Germany). RNA quantity was assessed using fluorometric quantification, and quality was evaluated using a Fragment Analyzer (Agilent, Santa Clara, CA, USA). Library preparation and sequencing were performed at the Mellowes Center. 500 ng of high-quality total RNA was input for the stranded total RNA library preparation protocol (Illumina). Library quality control was assessed using Kapa Quantification and a MiSeq (Roche Diagnostics, Pleasanton, CA, USA) 50-cycle run. Sequencing was performed on the Illumina NovaSeq 6000 with paired-end reads of 2 × 100 bp, targeting >50 million reads per sample. The sequencing data were aligned to the GRCh38.p14 genome assembly. Raw sequencing reads were processed through the MAP-RSeq v3.0 [20] bioinformatics workflow. Raw and normalized (RPKM) counts for 19,994 genes and corresponding exons. The R package edgeR v3.8.6 [21] was used for differential analysis of gene expression comparing each construct post-induction (+doxy) to the Empty vector (+doxy) in triplicate. Genes were considered sufficiently expressed for analysis if they showed counts per million (CPM) > 1 in at least 2 samples. Gene set enrichment analysis (GSEA) was subsequently conducted in Partek Flow, with pathways considered significant at *p* < 0.05. Additional differential expression analysis was performed using DESeq2. Genes with a false discovery rate below 0.05 and an absolute log2 fold change of at least plus or minus 0.75 were classified as significantly differentially expressed. We then further subsetted these genes based on Gene Ontology cellular component annotations, focusing specifically on nuclear membrane, nucleolus, and chromatin remodeling categories. We applied these expression thresholds to capture enhanced transcriptional heterogeneity between mutant conditions and to enable direct comparison of expression directionality across mutants. This approach allowed us to assess whether nuclear gene markers exhibited concordant expression trends across mutants or instead displayed distinct and opposing expression patterns, thereby highlighting differential nuclear regulatory states associated with each mutant background. To quantify nuclear transcriptional remodeling across KRAS variants, we calculated the Nuclear Transcriptional Remodeling Score (NTRS) as the arithmetic mean of the log2 fold change values for nuclear-associated genes within each condition. Upregulated and downregulated genes were analyzed separately by computing the mean log2 fold change for genes with positive and negative values, respectively. All genes meeting the predefined nuclear annotation criteria were weighted equally, with no additional scaling or ranking applied. The NTRS was used as a descriptive measurement metric and was not subjected to statistical testing.

### 4.4. Protein Phosphorylation Profiling

Protein phosphorylation was profiled using the PhosphoExplorer antibody array (Full Moon BioSystems, Sunnyvale, CA, USA; PEX100), which contains 1318 antibodies covering 584 phosphorylation sites across 452 signaling proteins, with each antibody represented in duplicate. Arrays were processed according to the manufacturer’s instructions. HPNE cells expressing nine KRAS mutants (G12C, G12D, G12V, G12R, G13D, Q61H, Q61K, Q61R, and the dominant-negative S17N) were analyzed at baseline (no mutant KRAS expression, −doxy) and after 24 h of doxycycline-induced KRAS expression (+doxy), with three biological replicates per condition. Signal intensities were quantified using the average median value for each antibody and normalized to the global median signal. For each phosphorylation site, a signal ratio was calculated by dividing the phosphorylated signal by the corresponding non-phosphorylated signal. Fold change values were then computed by comparing +doxycycline samples to their matched no doxycycline (−doxy) controls. Phosphorylation events were considered differentially regulated if they exhibited a log2 fold change ≥ 1 or ≤−1. No formal statistical significance testing was applied to the phosphorylation data; differentially regulated sites were defined based on log2 fold change thresholds (≥1 or ≤−1).

### 4.5. Immunofluorescence Microscopy

HPNE cells were cultured on glass coverslips and treated with doxycycline for 0, 24, or 48 h. Cells were fixed with 0.4% paraformaldehyde in PBS for 10 min, permeabilized with 0.3% Triton X-100 in PBS for 1 h, and blocked in 2% BSA in PBS for 1 h at room temperature. Primary antibodies diluted in 2% BSA containing 0.3% Triton X-100 were applied overnight at 4 °C. After washing, cells were incubated with secondary antibodies diluted in 2% BSA for 1 h at room temperature. Coverslips were mounted using antifade mounting medium and sealed prior to imaging. Primary antibodies were used at a 1:1000 dilution and included anti-nucleolin (Abcam, Cambridge, UK, ab22758), anti-fibrillarin [38F3] (Abcam, ab4566), and anti-SC-35 (Abcam, ab11826). Nuclei were counterstained with DAPI (Thermo Fisher Scientific, Waltham, MA, USA, D3571). Secondary antibodies were used at 1:1000 and included goat anti-mouse IgG (H+L) Alexa Fluor Plus 488 (Thermo Fisher Scientific, A32723) and goat anti-rabbit IgG (H+L) Alexa Fluor 555 (Thermo Fisher Scientific, A-21428). Two-dimensional fluorescence imaging was performed using a Keyence BZ-X800 microscope (Keyence Corporation, Osaka, Japan) (version 01.02.03.02) with a 60× objective at a resolution of 1920 × 1440 pixels. Images were acquired in triplicate, with each replicate containing 30–50 cells per condition. Quantitative analysis of 2D datasets was performed using QuPath v0.6.0, with automated cell and nuclear masking to ensure consistent, unbiased quantification. Three-dimensional confocal imaging was performed using an Andor Dragonfly 620SR multimodal spinning disk (Oxford Instruments, Belfast, UK) confocal system with a 60× objective at a resolution of 2048 × 2048 pixels (Fusion version 2.6.0; z-step size = 0.03 µm). Confocal datasets were acquired in triplicate, with each replicate containing 80–100 cells per condition. Image reconstruction, quantitative analyses, and automated masking were performed using Imaris (Oxford Instruments/Bitplane Zurich, Switzerland) version 11.0. Quantitative measurements derived from QuPath version 0.6.0 (University of Edinburgh (Edinburgh, UK)) and Imaris were analyzed in GraphPad Prism (GraphPad Software, San Diego, CA, USA) version 11.0.0. Statistical significance across conditions was assessed using a paired one-way analysis of variance (ANOVA) with Sidak’s multiple comparisons test to correct for multiple pairwise comparisons relative to the 0 h (−doxy) controls. Imaging acquisition and quantitative analyses were performed with knowledge of the experimental condition and were not conducted in a blinded manner.

### 4.6. STED Super-Resolution Microscopy

Three-dimensional stimulated emission depletion (3D-STED) super-resolution imaging was performed using a Facility Line STED system (Abberior, Göttingen, Germany) with a voxel size of 30 nm × 30 nm × 65 nm. Cells were prepared as described above for immunofluorescence microscopy. Primary antibodies were used at a dilution of 1:500 and included anti-nucleolin (Abcam, ab22758), anti-fibrillarin [38F3] (Abcam, ab4566), and anti-SC-35 (Abcam, ab11826). For SC-35 staining, nuclei were co-stained with anti-CTCF (D31H2) XP (Cell Signaling Technology, Danvers, MA, USA, 3418S). Secondary antibodies included Abberior STAR RED goat anti-rabbit IgG (STRED-1002-500UG) and Abberior STAR ORANGE goat anti-mouse IgG (STORANGE-1001-500UG), each used at 1:500. Imaging was performed using a 775 nm STED depletion laser controlled by Lightbox (Abberior) software version 2024.8.19704. STED imaging was performed in duplicate, with each replicate comprising 3–5 individual cells per condition. Cells were selected from fields representative of the nuclear and subnuclear features quantified in the corresponding 2D and 3D confocal analyses. For super-resolution image quantification, signal detection thresholds were defined using a single parameter set derived from baseline (0-h) samples and applied uniformly across all KRAS variants and time points. Threshold values were established based on signal-to-background intensity distributions and localization density metrics to exclude background noise while preserving biologically relevant structures. All segmentation and quantification steps were performed using identical analysis pipelines to ensure direct comparability of nuclear and subnuclear features across experimental conditions.

## Data Availability

Raw and processed data for the current study are available through Additional File S1 and Gene Expression Omnibus under accession GSE230860.

## Supplementary Materials

The following supporting information can be downloaded at: https://www.mdpi.com/article/10.3390/epigenomes10010019/s1, Figure S1: Validation of KRAS expression and specificity of RAS family transcriptional regulation (A) Western blot analysis showing comparable KRAS protein expression across HPNE cells expressing different KRAS mutant variants following doxycycline induction, relative to non-induced wild type HPNE control cells. (B) Transcriptomic profiling of RAS family members demonstrating induced KRAS expression across all KRAS mutant conditions, with HRAS and NRAS expression remaining unchanged relative to non-induced controls. (C) Nuclear Transcriptional Remodeling Score (NTRS) summarizing bidirectional nuclear transcriptional responses downstream of each KRAS variant, calculated as the mean log2 fold change in upregulated and downregulated nuclear-associated genes relative to non-induced wild-type controls.; Table S1: RNA sequencing analysis of nuclear-associated transcripts across KRAS mutant variants. Additional File S1: Transcriptomic and phospho-proteomic datasets for KRAS variant–dependent nuclear transcriptional remodeling.

## Abbreviations

The following abbreviations are used in this manuscript:

PDAC	Pancreatic ductal adenocarcinoma
GSEA	Gene set enrichment analysis
KEGG	Kyoto Encyclopedia of Genes and Genomes
HPNE	Human pancreatic nestin-expressing (cells)
NTRS	Nuclear Transcriptional Remodeling Score
DAPI	4′,6-diamidino-2-phenylindole
STED	Stimulated emission depletion

## References

[1] Bahl S., Seto E. (2020). Regulation of Histone Deacetylase Activities and Functions by Phosphorylation and Its Physiological Relevance. Cell. Mol. Life Sci..

[2] Adenuga D., Rahman I. (2010). Protein Kinase CK2-Mediated Phosphorylation of HDAC2 Regulates Co-Repressor Formation, Deacetylase Activity and Acetylation of HDAC2 by Cigarette Smoke and Aldehydes. Arch. Biochem. Biophys..

[3] Xing Z., Ma W.K., Tran E.J. (2019). The DDX5/Dbp2 Subfamily of DEAD-Box RNA Helicases. Wiley Interdiscip. Rev. RNA.

[4] Loughery J., Cox M., Smith L.M., Meek D.W. (2014). Critical Role for P53-Serine 15 Phosphorylation in Stimulating Transactivation at P53-Responsive Promoters. Nucleic Acids Res..

[5] Mathison A.J., Kerketta R., de Assuncao T.M., Leverence E., Zeighami A., Urrutia G., Stodola T.J., di Magliano M.P., Iovanna J.L., Zimmermann M.T. (2021). KrasG12D Induces Changes in Chromatin Territories That Differentially Impact Early Nuclear Reprogramming in Pancreatic Cells. Genome Biol..

[6] Flores L.F., Marks D.L., Vera R.E., Sigafoos A.N., Tolosa E.J., Almada L.L., Pease D.R., Toruner M.D., Chang B., Tader B.R. (2025). Emerin Is an Effector of Oncogenic KRAS-Driven Nuclear Dynamics in Pancreatic Cancer. JCI Insight.

[7] Yuan T.L., Amzallag A., Bagni R., Yi M., Afghani S., Burgan W., Fer N., Garvey L., Powell K., Smith B. (2018). Differential Effector Engagement by Oncogenic KRAS. Cell Rep..

[8] Dubois M.-L., Boisvert F.-M. (2016). The Nucleolus: Structure and Function. The Functional Nucleus.

[9] Azman M.S., Alard E.L., Dodel M., Capraro F., Faraway R., Dermit M., Fan W., Chakraborty A., Ule J., Mardakheh F.K. (2023). An ERK1/2-Driven RNA-Binding Switch in Nucleolin Drives Ribosome Biogenesis and Pancreatic Tumorigenesis Downstream of RAS Oncogene. EMBO J..

[10] Yousef A., Yousef M., Chowdhury S., Abdilleh K., Knafl M., Edelkamp P., Alfaro-Munoz K., Chacko R., Peterson J., Smaglo B.G. (2024). Impact of KRAS Mutations and Co-Mutations on Clinical Outcomes in Pancreatic Ductal Adenocarcinoma. npj Precis. Oncol..

[11] Nusrat F., Khanna A., Jain A., Jiang W., Lavu H., Yeo C.J., Bowne W., Nevler A. (2024). The Clinical Implications of KRAS Mutations and Variant Allele Frequencies in Pancreatic Ductal Adenocarcinoma. J. Clin. Med..

[12] Hobbs G.A., Baker N.M., Miermont A.M., Thurman R.D., Pierobon M., Tran T.H., Anderson A.O., Waters A.M., Diehl J.N., Papke B. (2020). Atypical KRASG12R Mutant Is Impaired in PI3K Signaling and Macropinocytosis in Pancreatic Cancer. Cancer Discov..

[13] Takada H., Kurisaki A. (2015). Emerging Roles of Nucleolar and Ribosomal Proteins in Cancer, Development, and Aging. Cell. Mol. Life Sci..

[14] Faber G.P., Nadav-Eliyahu S., Shav-Tal Y. (2022). Nuclear Speckles—A Driving Force in Gene Expression. J. Cell Sci..

[15] Lo A., McSharry M., Berger A.H. (2022). Oncogenic KRAS Alters Splicing Factor Phosphorylation and Alternative Splicing in Lung Cancer. BMC Cancer.

[16] Sobhani N., Pittacolo M., D’Angelo A., Marchegiani G. (2025). Recent Anti-KRASG12D Therapies: A “Possible Impossibility” for Pancreatic Ductal Adenocarcinoma. Cancers.

[17] Ouyang H., Mou L., Luk C., Liu N., Karaskova J., Squire J., Tsao M.-S. (2000). Immortal Human Pancreatic Duct Epithelial Cell Lines with Near Normal Genotype and Phenotype. Am. J. Pathol..

[18] Tripathi K., Parnaik V.K. (2008). Differential Dynamics of Splicing Factor SC35 during the Cell Cycle. J. Biosci..

[19] Noel J.S., Dewey W.C., Abel J.H., Thompson R.P. (1971). Ultrastructure of the Nucleolus During the Chinese Hamster Cell Cycle. J. Cell Biol..

[20] Kalari K.R., Nair A.A., Bhavsar J.D., O’Brien D.R., Davila J.I., Bockol M.A., Nie J., Tang X., Baheti S., Doughty J.B. (2014). MAP-RSeq: Mayo Analysis Pipeline for RNA Sequencing. BMC Bioinform..

[21] Robinson M.D., McCarthy D.J., Smyth G.K. (2010). edgeR: A Bioconductor Package for Differential Expression Analysis of Digital Gene Expression Data. Bioinformatics.

