# Supplementary material for: Mutant KRAS Heterogeneity Shapes Nuclear Architecture During Pancreatic Cancer Initiation
Source: Epigenomes. 2026 Mar 10;10(1):19. doi: 10.3390/epigenomes10010019 (PMC13025234; doi:10.3390/epigenomes10010019)
Supplement: Supplementary file 1 [file epigenomes-10-00019-s001.zip › Pollin_etal_HPNE_2026_Table_S1 Review.pdf]

Table S1: RNA sequencing analysis of nuclear-associated transcripts across KRAS mutant variants. RNA-seq-derived log2 fold change values for a curated set of nuclear-associated genes annotated by Gene Ontology cellular component terms, including chromatin remodeling, nuclear membrane, and nucleolar categories.

**Gene**	Component	**WT**	**G12C**	**G12D**	**G12R**	**G12V**	**G13D**	**Q61H**	**Q61K**	**Q61R**	**S17N**
ADRA1B	Nuclear Membrane	0.43	0.25	-0.56	0.10	-0.22	-0.11	-0.90	-0.29	-0.21	1.88
BCL2	Nuclear Membrane	1.29	1.57	0.36	0.51	1.08	1.15	0.38	0.93	0.70	0.94
CEMIP	Nuclear Membrane	1.39	1.51	0.24	0.22	0.54	0.91	-0.75	0.69	-0.40	1.69
DMPK	Nuclear Membrane	-0.11	-0.21	-0.57	0.18	-0.36	-0.20	-0.62	-0.47	-0.39	1.64
EPHA3	Nuclear Membrane	-0.99	-1.08	-0.74	-0.40	-1.34	-1.29	-0.52	-1.19	-0.92	0.10
GATA6	Nuclear Membrane	1.01	1.04	0.16	0.61	0.27	0.62	-1.04	-0.13	-1.22	2.45
GCHFR	Nuclear Membrane	-0.52	-0.87	-0.90	-0.13	-1.40	-0.82	-1.46	-1.56	-0.51	1.16
IFI27	Nuclear Membrane	-0.14	-0.37	-0.01	0.47	-0.43	-0.92	-0.71	-0.37	-0.09	-0.22
NOS1AP	Nuclear Membrane	1.22	2.06	2.06	0.84	2.11	2.01	2.19	2.61	1.90	-0.66
PRICKLE1	Nuclear Membrane	0.36	0.86	0.93	-0.01	1.08	0.57	0.87	1.41	0.86	1.28
PTGS2	Nuclear Membrane	0.29	0.39	1.05	-0.10	0.60	0.05	0.64	0.22	0.36	-0.24
SMAD3	Nuclear Membrane	-0.27	-0.72	-0.92	-0.04	-0.82	-0.60	-0.73	-1.02	-0.93	0.07
SYNE1	Nuclear Membrane	-0.20	-0.56	-0.44	-0.40	-0.52	-0.59	-0.19	-0.68	-0.91	0.20
SYNE2	Nuclear Membrane	-0.14	-0.59	-0.41	-0.53	-0.49	-0.76	-0.20	-0.77	-0.96	0.40
TM7SF2	Nuclear Membrane	-0.81	-1.34	-0.73	-0.04	-0.75	-1.16	-0.52	-1.32	-0.16	-0.35
TNRC18	Nuclear Membrane	0.13	-0.11	-0.36	0.12	-0.31	0.03	-0.10	-0.20	-0.30	1.09
ABTB1	Nucleolus	-0.04	-0.37	-1.24	0.03	-0.80	-0.43	-1.00	-0.78	-0.78	0.59
ANG	Nucleolus	0.41	0.29	-0.38	0.21	0.11	0.24	-0.33	0.12	-0.03	1.08
ATF3	Nucleolus	0.19	0.05	0.23	0.20	-0.18	-0.18	-0.72	-0.39	-0.59	1.04
ATXN1	Nucleolus	-0.25	-0.48	-0.47	-0.05	-0.36	-0.58	-0.35	-0.62	-0.81	0.63
BOP1	Nucleolus	0.39	0.83	0.78	0.45	0.82	0.83	0.69	1.03	1.00	0.30
CCDC86	Nucleolus	0.43	0.85	0.89	0.30	0.82	0.75	0.79	1.03	0.99	0.11
CDC6	Nucleolus	0.04	0.06	0.30	-0.16	0.27	0.17	0.31	0.20	0.36	-1.04
CHRM2	Nucleolus	1.02	0.58	-0.89	-0.12	-0.59	0.16	-1.48	-0.23	-1.77	2.04
DOCK4	Nucleolus	-0.48	0.00	0.68	-0.10	0.45	0.18	0.91	0.55	0.38	-1.74
ETV4	Nucleolus	-0.14	-0.16	0.11	0.31	-0.03	0.16	0.39	0.09	0.48	-1.35
GLI3	Nucleolus	0.01	-0.21	-0.47	-0.12	-0.65	-0.28	-0.37	-0.50	-0.84	0.08
GPER1	Nucleolus	-1.03	-0.91	0.00	-0.04	-0.01	-0.46	0.23	-0.24	0.35	-0.98
IFI35	Nucleolus	-0.44	-0.69	-0.70	0.26	-0.71	-0.51	-0.82	-0.87	-0.45	0.32
IGF1R	Nucleolus	0.22	0.09	-0.53	-0.12	-0.29	-0.09	-0.48	-0.31	-0.81	0.70
L3MBTL1	Nucleolus	-0.38	-0.67	-0.52	-0.17	-0.88	-0.60	-0.53	-0.90	-0.78	0.20
MTUS1	Nucleolus	-0.82	-1.65	-1.83	0.23	-1.63	-1.36	-1.02	-2.15	-1.26	-1.09
MXI1	Nucleolus	-0.28	-0.44	-0.86	0.06	-0.70	-0.41	-0.49	-0.76	-0.61	0.61
NOP53	Nucleolus	0.33	0.29	-0.01	0.48	0.29	0.52	0.02	0.26	0.38	1.21
PODXL	Nucleolus	-0.17	0.00	0.69	-0.41	0.09	0.04	0.60	0.02	-0.08	-0.99
POLA1	Nucleolus	-0.05	-0.14	0.17	-0.22	0.23	-0.06	0.26	0.08	0.15	-0.92

PRDM1	Nucleolus	0.47	0.99	1.09	0.11	0.96	0.88	0.90	1.32	0.70	-0.11
RASL11A	Nucleolus	0.43	0.66	0.23	0.65	0.16	0.51	-0.28	0.37	0.29	2.86
RCAN2	Nucleolus	-0.62	-1.34	-1.21	-0.34	-1.04	-0.86	-0.71	-1.40	-0.62	-0.18
RELT	Nucleolus	-0.36	-0.09	0.25	-0.09	-0.10	-0.33	0.27	-0.15	0.16	-0.81
RNF213	Nucleolus	-0.02	-0.23	-0.33	-0.25	-0.38	-0.26	-0.21	-0.43	-0.87	0.25
RRS1	Nucleolus	0.47	0.82	0.83	0.36	0.91	0.74	0.91	1.05	1.09	0.27
SELENBP1	Nucleolus	-0.93	-1.41	-0.92	-0.09	-0.84	-0.91	-0.85	-1.37	-0.63	0.00
TP53	Nucleolus	-0.19	-0.50	-0.89	-0.10	-0.92	-0.50	-0.80	-0.72	-0.90	0.19
TWIST2	Nucleolus	0.42	1.03	0.81	0.69	1.07	1.44	0.93	1.23	1.15	0.40
UBXN8	Nucleolus	-0.28	-0.34	0.25	0.11	0.06	-0.08	0.35	0.20	0.49	-0.88
APOBEC3F	Chromatin Remodelling	-0.46	-0.80	-0.31	-0.02	-0.59	-0.60	-0.58	-0.88	-0.33	-0.19
CHD6	Chromatin Remodelling	-0.01	-0.33	-0.62	-0.35	-0.48	-0.40	-0.39	-0.49	-0.87	0.20
EGR1	Chromatin Remodelling	-0.43	-0.19	0.41	0.33	0.31	0.18	0.71	0.51	0.69	-2.81
GDNF	Chromatin Remodelling	1.08	1.37	0.67	0.19	0.78	0.88	0.04	0.97	0.13	1.09
HDAC5	Chromatin Remodelling	0.18	-0.02	-0.73	0.16	-0.61	-0.14	-0.90	-0.49	-0.81	1.73
HDAC9	Chromatin Remodelling	-0.20	-0.46	-0.43	-0.58	-0.70	-0.85	-0.61	-0.86	-1.08	0.15
MYOCD	Chromatin Remodelling	0.52	0.04	-0.24	-0.18	-0.34	-0.46	-1.07	-0.48	-1.21	1.80
PWWP2B	Chromatin Remodelling	0.23	0.35	-0.01	0.61	0.23	0.40	0.33	0.33	0.34	1.51
SATB1	Chromatin Remodelling	-0.44	-0.73	-1.13	-0.05	-0.88	-0.80	-0.75	-1.10	-1.09	0.32
SLFN11	Chromatin Remodelling	-0.19	-0.28	0.11	-0.13	-0.06	-0.18	0.25	0.00	0.01	-0.82
SNAI1	Chromatin Remodelling	0.00	0.05	0.06	0.45	-0.05	-0.11	0.09	0.20	0.30	1.56
SOX9	Chromatin Remodelling	0.74	1.81	1.47	0.85	1.66	1.84	1.44	2.13	1.61	-0.05
TCF7L1	Chromatin Remodelling	0.05	-0.30	-0.59	-0.34	-0.46	-0.50	-0.62	-0.57	-0.94	1.30
TET1	Chromatin Remodelling	0.17	0.09	-0.18	-0.20	-0.03	-0.30	-0.18	-0.05	-0.91	1.19
