# Supplementary figures and images for: Mutant KRAS Heterogeneity Shapes Nuclear Architecture During Pancreatic Cancer Initiation
Source: Epigenomes. 2026 Mar 10;10(1):19. doi: 10.3390/epigenomes10010019 (PMC13025234; doi:10.3390/epigenomes10010019)

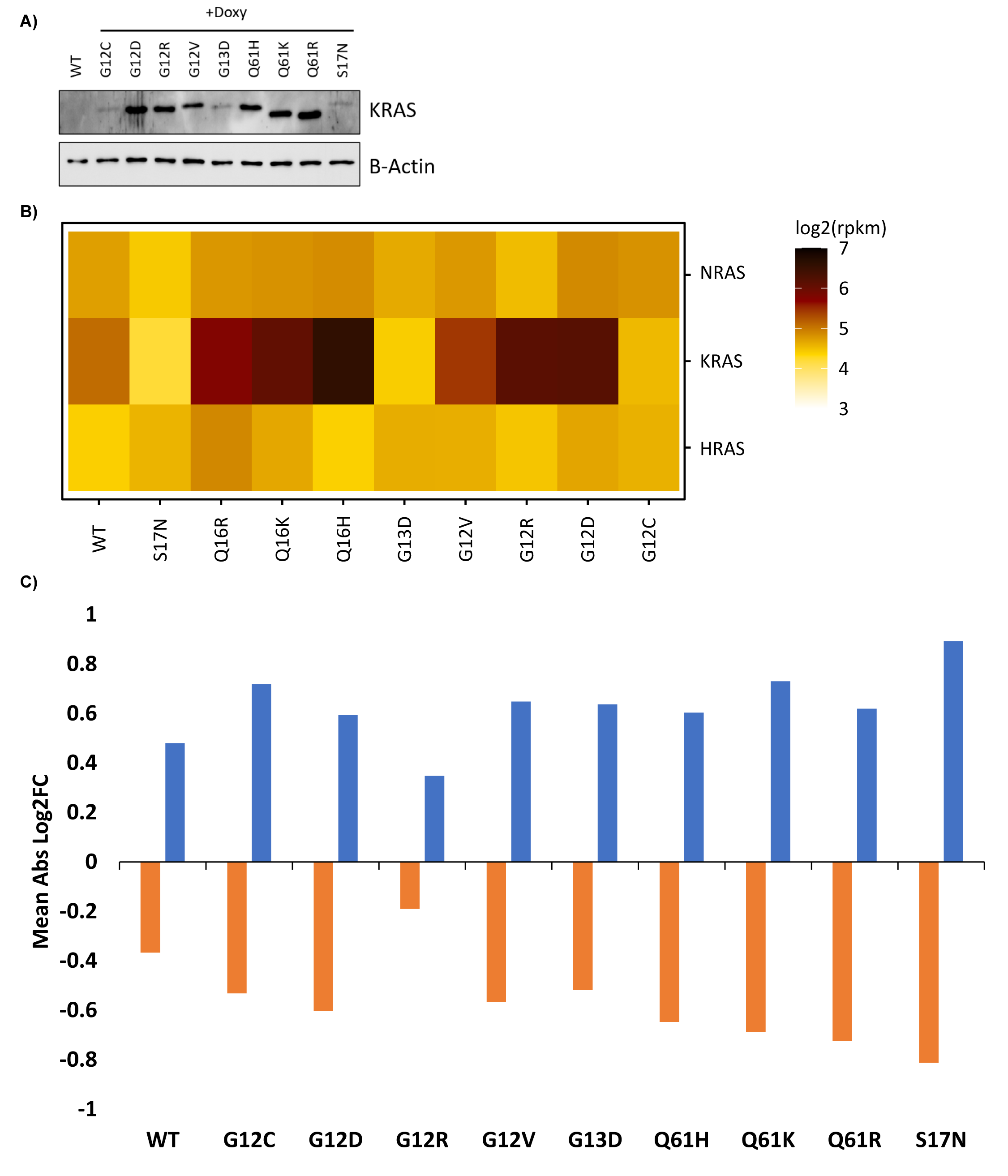

Supplement: Supplementary file 1 [file epigenomes-10-00019-s001.zip › Supplementary Figure S1.tif]
